# Cross-Sample Validation Provides Enhanced Proteome Coverage in Rat Vocal Fold Mucosa

**DOI:** 10.1371/journal.pone.0017754

**Published:** 2011-03-15

**Authors:** Nathan V. Welham, Masaru Yamashita, Seong Hee Choi, Changying Ling

**Affiliations:** Department of Surgery, Division of Otolaryngology, University of Wisconsin School of Medicine and Public Health, Madison, Wisconsin, United States of America; St. Georges University of London, United Kingdom

## Abstract

The vocal fold mucosa is a biomechanically unique tissue comprised of a densely cellular epithelium, superficial to an extracellular matrix (ECM)-rich lamina propria. Such ECM-rich tissues are challenging to analyze using proteomic assays, primarily due to extensive crosslinking and glycosylation of the majority of high *M*_r_ ECM proteins. In this study, we implemented an LC-MS/MS-based strategy to characterize the rat vocal fold mucosa proteome. Our sample preparation protocol successfully solubilized both proteins and certain high *M*_r_ glycoconjugates and resulted in the identification of hundreds of mucosal proteins. A straightforward approach to the treatment of protein identifications attributed to single peptide hits allowed the retention of potentially important low abundance identifications (validated by a cross-sample match and *de novo* interpretation of relevant spectra) while still eliminating potentially spurious identifications (global single peptide hits with no cross-sample match). The resulting vocal fold mucosa proteome was characterized by a wide range of cellular and extracellular proteins spanning 12 functional categories.

## Introduction

The vocal fold (VF) mucosa is a complex multi-layered biological system consisting of a squamous cell epithelium, basement membrane and lamina propria (LP). Each mucosal layer holds a distinct set of functions that are together responsible for VF immune, transport and barrier capabilities, the ability to absorb considerable impact stress, and favorable viscoelasticity for self-sustained tissue oscillation and voice production [1]–[9]. The epithelium and basement membrane represent the most superficial layers of the VF mucosa and jointly provide a protective physical barrier against mucosal insult [1], [3]. Surface epithelial cells signal professional immune cells in response to incident challenges from the upper airway [7], [8], [10], [11] and mediate water and ion transport for the maintenance of VF surface hydration [4]–[6].

Deep to the basement membrane, the LP is populated by sparsely distributed fibroblast cells housed in a biomechanically favorable extracellular matrix (ECM) [2], [9]. ECM fibrous proteins (collagens and elastins) confer three-dimensional matrix organization, strength and elasticity [2]; whereas interstitial glycans (proteoglycans, glycoproteins and glycosaminoglycans) influence matrix viscosity, hydration and volume [9]. These proteins and glycans are functionally interdependent within the ECM, and often operate in a synchronous and coordinated fashion. For example, decorin modulates stress transmission along collagen fibrils, and also influences fibril organization; fibromodulin binds to collagen and regulates collagen synthesis; fibronectin facilitates cell adhesion and upregulates collagen at wound sites; and versican binds to hyaluronic acid, allows compression, and dissipates impact stress [9], [12]–[14]. These coordinated interactions underscore the inherent complexity of both ECM and overall VF mucosal function as well as the importance of investigating complete functional protein-protein and protein-glycan groups using system-wide methodologies.

While the importance of the VF mucosa (and its protein/glycan constituents) to overall VF physiology and voice production is clear [2], [9], scientific understanding of its native biological structure and function, and the manner in which it is altered under certain physiological and disease states, remains limited. Historically, most VF research has been driven by an experimental paradigm focused on individual and small groups of genes/proteins, selected based on their presumed structure and function, and generally informed by work conducted in other mucosal systems. These approaches have generated improved appreciation of specific mucosal constituents, but hold notable limitation in contributing to an overarching and unifying understanding of how these individual players interact to form a functional biological and biomechanical system. Microarrays and other mRNA detection technologies have given insight into the transcriptome-wide regulation of diseased VF mucosa [15]; however these assays do not address important parameters such as alternately spliced transcripts and post-translational modifications. Proteomic datasets transcend these limitations by capturing the operational profiles of the majority of expressed proteins subsequent to transcription and translation, and in doing so represent the entire functional output of a given system. As such, proteomic approaches promise to alter how the VF mucosa is conceptualized and potentially open new avenues in the evaluation and treatment of VF mucosal disease.

ECM-rich tissues such as the VF mucosa are challenging to analyze using proteomic assays, primarily due to the extensive crosslinking and glycosylation of many high *M*_r_ ECM proteins [16], [17]. In this study, we implemented an LC-MS/MS-based strategy to characterize the rat VF mucosa proteome. The rat is a well-accepted model in VF biology [18]–[24] and has been previously used in proteomic studies of the thyroarytenoid (TA) muscle [25]–[27]. We successfully solubilized both proteins and high *M*_r_ glycoconjugates from rat VF mucosa, and identified a comprehensive library of proteins spanning twelve functional categories.

## Results

**Figure 1:**
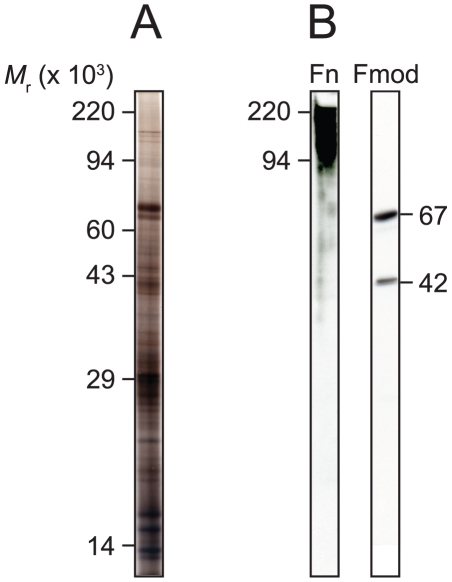
1-DE of rat vocal fold mucosa demonstrates high sample complexity and ECM glycoprotein/proteoglycan retention. (A) Representative sample separated by 10% SDS-PAGE and silver stained. (B) Representative immunoblots for fibronectin (Fn) and fibromodulin (Fmod). Fibronectin was detected at 220×10^3^ *M*_r_ and appeared variably glycosylated. Fibromodulin was detected as two distinct bands at 42×10^3^ *M*_r_ (non-glycosylated core protein) and 67×10^3^ *M*_r_ (N-linked oligosaccharide substituted form).

We first evaluated our ability to solubilize proteins and high *M*_r_ glycoconjugates from rat VF mucosa samples. Fig. 1 illustrates representative 1-DE separation and positive immunoblotting of rat VF mucosa for the glycoprotein fibronectin and proteoglycan fibromodulin, confirming successful extraction and retention of these glycosylated ECM constituents. Fibronectin was detected at an expected 220×10^3^ *M*_r_ (native fibronectin is comprised of two 220×10^3^ *M*_r_ subunits which are separated on reducing SDS-PAGE) and appeared as a diffuse band suggesting varying degrees of glycosylation. Fibromodulin was detected as two distinct bands at 42 and 67×10^3^ *M*_r_. Based on previous electrophoretic characterization [28], [29], the 42×10^3^ *M*_r_ band is consistent with the non-glycosylated fibromodulin core protein and the 67×10^3^ *M*_r_ band is consistent with its N-linked oligosaccharide-substituted form. We did not observe evidence for a keratan sulfate-substituted form (typically detected as a series of diffuse bands between 70 and 110×10^3^ *M*_r_) in these samples.

Next, we performed parallel LC-MS/MS runs on three independent samples, following initial *M*_r_-based sample fractionation using 1-DE. Peptide and protein identifications were compared across independent sample runs in an attempt to salvage and validate potentially important low abundance proteins, as follows. Cross-sample matching was performed with special consideration of proteins identified by a single unique peptide. Proteins identified by a single unique peptide in a given sample (termed *local single peptide hits*) were categorized into two subsets: Those with a corresponding protein match in another sample (such a cross-sample match could have any number of peptide hits), and those with no corresponding protein match in another sample (termed *global single peptide hits*). Matching of protein identifications across samples was then performed with all peptide hits retained, with local single peptide hits removed, and with global single peptide hits removed.

**Figure 2:**
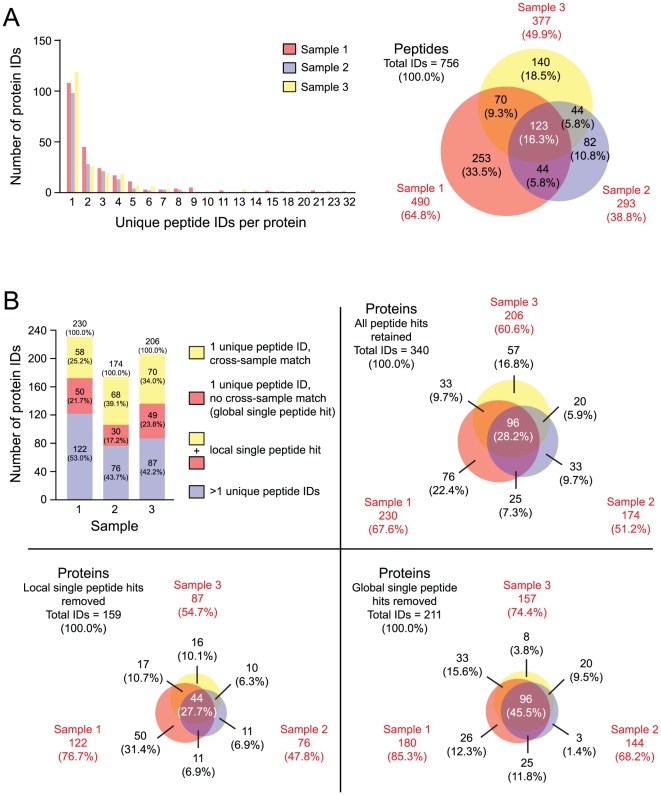
Comparison of peptide and protein identifications generated from LC-MS/MS runs representing three independent vocal fold mucosa samples. (A) Distribution of number of unique peptides per protein identification across samples, and overlap in unique peptide identifications across samples. (B) Number of protein identifications and overlap across samples, with varying treatment of proteins identified by a single unique peptide identification (all peptide hits retained, local single peptide hits removed, global single peptide hits removed). Due to rounding, not all percentages total to 100.0%.

We initially identified a total of 756 unique peptides associated with 340 proteins across all three samples, using a 1% estimated false discovery rate (Fig. 2A–B). This analysis was marked by a significant number of local single peptide hits (108 [46.9% of 230] in sample 1; 98 [56.3% of 174] in sample 2; 119 [57.8% of 206] in sample 3). Removing all local single peptide hits prior to matching resulted in a 53.2% decrease in total proteins identified to 159, whereas removing only global single peptide hits resulted in a 37.9% decrease in total proteins identified to 211 (Fig. 2B). Further, as the removal of global single peptide hits only affected protein identifications with no cross-sample matches, this strategy yielded improved percentage agreement across samples, resulting in 82.5% of identified proteins matched across at least two of three samples (Fig. 2B).

**Figure 3:**
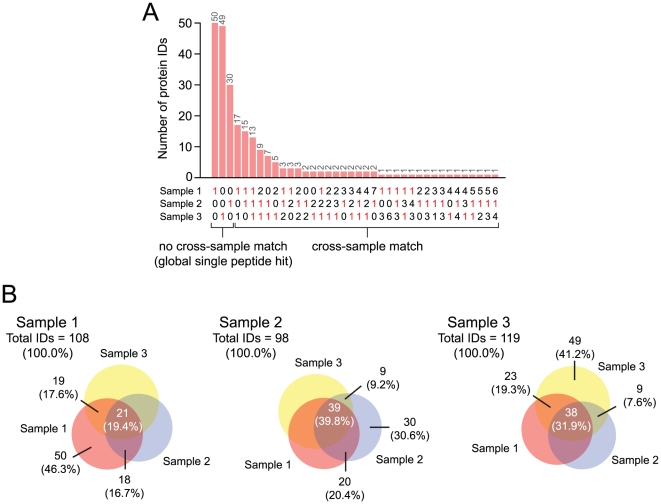
Analysis of proteins identified by a single unique peptide in LC-MS/MS runs representing three independent vocal fold mucosa samples. (A) Distribution of protein identifications with a single unique peptide hit in one or more samples (local single peptide hits). Data are further subcategorized into proteins with no cross-sample match (global single peptide hits) and proteins with a cross-sample match. (B) Venn diagrams illustrating overlap in local single peptide hits (i.e., number of cross-sample matches) for each sample. Non-overlapping regions in each Venn diagram represent global single peptide hits. Due to rounding, not all percentages total to 100.0%.

Detailed analysis of local single peptide hits (Fig. 3A–B) revealed that 30.6–46.3% of these protein identifications were global single peptide hits, confirming that the majority of protein identifications associated with a single unique peptide had a positive cross-sample match. Further, 19.4-39.8% of these identifications were matched across all three samples (Fig. 3B). A large number of cross-sample matches were to other single unique peptides; however, some matches had as many as seven unique peptides (Fig. 3A). To complement this analysis, we implemented secondary validation of MS/MS spectra associated with local single peptide hits using *de novo* peptide sequencing followed by MS-driven BLAST searching [30]. Thirty-two database hits failed this validation step and were therefore considered false positives.

Table S1 contains functional classification data for proteins identified by LC-MS/MS following the removal of global single peptide hits and local single peptide hits derived from spectra that failed *de novo* sequencing-based validation. Proteins were classified using annotation and categorization data in the UniProtKB/Swiss-Prot database [31]. A wide range of cellular and extracellular proteins were identified, spanning 12 functional categories: Circulatory system, blood proteins; cytoskeletal proteins (microfilament, intermediate filament, microtubules) including nuclear envelope and epithelial keratins; DNA binding proteins; defense, stress and immune response proteins; ECM proteins; membrane (cell, nuclear, mitochondrial) proteins; metabolism and energy proteins; cell motility, contractile/thick filament proteins; protein fate (maturation, modification, trafficking, degradation); signaling proteins; protein translation/synthesis; and miscellaneous proteins.

**Figure 4:**
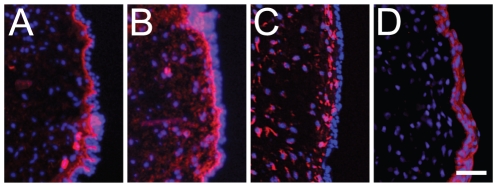
Representative images showing immunohistochemical validation of four vocal fold mucosa proteins identified using LC-MS/MS. Frozen sections were stained with antibodies anti-collagen type I (labeled red in A), anti-fibronectin (labeled red in B), anti-vimentin (labeled red in C), anti-keratin Ka10 (labeled red in D), and nuclear dye DAPI (labeled blue in A–D). Scale bar  = 40 µm.

We selected four representative VF mucosa proteins from Table S1 for additional immunohistochemical validation. The ECM protein collagen type I and glycoprotein fibronectin were detected throughout the LP, with preferential localization to the superficial LP (Fig. 4A–B). The intermediate filament protein vimentin was detected in the cytosol of the majority of cells in the LP (Fig. 4C); whereas the intermediate filament protein keratin Ka10 was exclusively localized to the epithelium (Fig. 4D).

## Discussion

The individual protein species and general categories identified in our dataset represent a wide array of structural and functional agents in the VF mucosa, many of which are of known importance to performance of this tissue, and therefore valuable markers for future quantitative proteomic studies. In addition to ubiquitous proteins that underpin fundamental cellular processes such as energy metabolism, transcription and translation, protein modification and transport, we identified a large complement of epithelial intermediate filament keratins, several ECM proteins and glycoconjugates, and a number of skeletal muscle thick filament proteins. Detection of these thick filament proteins infers muscle fiber contamination of our VF mucosa samples, despite careful microdissection and no evidence of TA muscle disturbance at the macro level. Complete elimination of all invasive muscle fibers may require preparation of frozen tissue sections followed by laser capture microdissection. This approach, which has been employed elsewhere in tissue proteomics [32], [33], would also allow accurate separation of the VF epithelium and LP, in addition to the investigation of regional areas of interest within the LP, such as the maculae flavae.

The tightly regulated protein/glycan constituency of the LP ECM is critical to the biomechanical capacity of the VF mucosa for self-sustained oscillation. In this study, we successfully extracted and identified a number of procollagen/collagen isoforms, in addition to the proteoglycans decorin and fibromodulin, and the glycoproteins fibronectin, fibrillin and laminin. It is important to note that a number of known LP ECM constituents (such as the fibrous proteins collagen type III and elastin, and glycosaminoglycan hyaluronic acid) were not detected in our LC-MS/MS runs. As noted, ECM is generally a challenging target for proteomic analysis due to the high *M*_r_, poor solubility and poor digestability of the majority of ECM proteins, many of which are extensively crosslinked and/or glycosylated [16], [17]. High *M*_r_ glycans and glycoconjugates are also known to impair isoelectric focusing during 2-DE [34]–[36]. Work in other ECM-rich tissues such as cartilage has shown improved protein resolution on 2-DE following depletion of high *M*_r_ glycans using centrifugal filtration [34], [37], [38], anion exchange chromatography [35] and cetylpyridinium chloride precipitation [36], [39]. Also, trypsin digestion of ECM prior to LC-MS/MS appears to be significantly enhanced by ultrasonication and incorporation of an acid-labile surfactant treatment [16]. Analysis of ECM glycans and glycoconjugates may be best achieved by initial isolation from the larger proteome using antibody or lectin affinity chromatography, and/or metabolic labeling [40]. Finally, compared to collision induced dissociation, electron transfer dissociation-based MS may be favorable for determining glycosylation site and glycan size, due its tendency to preferentially fragment the protein backbone while leaving glycan side chains largely intact [41].

The validation of borderline protein identifications using cross-sample matching of local single peptide hits in our dataset illustrates the value of performing MS/MS on multiple independent samples, and is a computationally straightforward approach to enhancing the identification of low abundance proteins. Further, secondary validation using *de novo* interpretation of relevant spectra provides additional protection against unwanted false positives. Conservative approaches to database-driven proteomics typically define a positive protein identification as characterized by two or more unique peptides [42]–[44]. Although this approach stringently guards against false positives, it also removes a large number of potentially valuable protein identifications (53% of total protein identifications in our dataset). The ideal management of single peptide hits involves maximizing true positive protein identifications while maintaining a strict false discovery rate. A recent body of literature in this area suggests that improved proteome coverage can be achieved by analyzing samples multiple times, using multiple MS instruments, and using multiple search algorithms [42]; and that true positive protein identifications associated with single peptide hits can be salvaged via *de novo* sequencing (as used in this study) [45], modified decoy database searching [46], and/or the application of various modeling approaches [43]. Interestingly, it appears that automatic elimination of all protein identifications based on single peptide hits results in the disproportionate depletion of positive identifications in target and decoy databases, which has driven a recent argument that protein identifications should be subject to estimated false-positive rates, similar to the current standard commonly employed for peptides [44]. The approach to managing single peptide hits employed in this study is attractive in that it maintains a stringent estimated false-positive rate at the peptide level, while salvaging a significant proportion of true positive protein identifications based on the assurance of cross-sample validation and *de novo* peptide sequencing.

## Materials and Methods

This study was performed in accordance with the PHS Policy on Humane Care and Use of Laboratory Animals, the NIH *Guide for the Care and Use of Laboratory Animals*, and the Animal Welfare Act (7 U.S.C. et seq.); the animal use protocol was approved by the Institutional Animal Care and Use Committee of the University of Wisconsin-Madison (approval M1742).

### Experimental animals

Three experimentally naïve four-month-old male Sprague Dawley rats were used for immunoblotting and proteomic assays. Each animal was euthanized via CO_2_ asphyxiation. The larynx was harvested *en bloc*, separated along the midline, and the VF mucosa (epithelium and entire LP) were undermined and dissected from the TA muscle. All dissection procedures were performed under a stereo dissection microscope using microsurgical instruments and 27-G needles. Each larynx was inspected to confirm TA muscle integrity following microdissection and each sample was processed for 1-D SDS-PAGE followed by either immunoblotting or band excision with subsequent LC-MS/MS. The time duration from euthanasia to completion of dissection was approximately 10 min in all cases.

### Sample preparation

VF mucosa samples (left and right samples from a single animal were combined) were placed in 25 µL osmotic lysis buffer (0.3% SDS, 10 mM Tris; pH 7.4) containing 10% nuclease (500 µg/mL RNase, 1 mg/mL DNase, 50 mM MgCl_2_, 100 mM Tris; pH 7.0) and 1% protease inhibitor (20 mM AEBSF, 1 mg/mL leupeptin, 360 µg/mL E-64, 500 mM EDTA, 560 µg/mL benzamidine) solutions. Tissue homogenization was performed on ice using an ultrasonic homogenizer (300V/T; Biologics, Manassas, VA) for 6 min at 40% power with a micro tip. After the addition of 25 µL boiling buffer (5% SDS, 10% glycerol, 60 mM Tris; pH 6.8), the samples were placed in a boiling water bath for 30 min to facilitate dissolution, cooled on ice, and then centrifuged to pellet solids. After removing an aliquot for total protein quantitation, the samples were microdialyzed at 4°C overnight using 5 mM Tris pH 6.8 and a 6–8×10^3^ *M*_r_ cut-off membrane filter. Next, the samples were lyophilized and reconstituted to 1 µg/µL in a 1∶1 ratio of boiling buffer to urea buffer (9.5 M urea, 2% w/v IGEPAL CA-630, 5% beta-mercaptoethanol) before gel loading.

Total protein quantitation was performed spectrophotometrically using the bicinchoninic acid method [47] and kit produced by Pierce Biotech (Rockford, IL). BSA was employed as a standard and absorbance at 562 nm was measured using the Smart Spec 3000 spectrophotometer (Bio-Rad, Hercules, CA). Samples were analyzed in duplicate and data were averaged. Mean final measurements of total protein were ∼150 µg for all samples.

### Electrophoresis

1-D SDS-PAGE was performed using a 0.75-mm thick 10% acrylamide slab gel. Electrophoresis was performed for approximately 4 h using 15 mA/gel. Total protein load was 5 µg for gels intended for silver staining [48] and 25 µg for a series of replicate gels intended for CBB staining, PVDF membrane transfer for immunoblotting, or band excision for LC-MS/MS. Six proteins (Sigma, St. Louis, MO) were employed as *M*_r_ standards: Myosin (220×10^3^), phosphorylase A (94×10^3^), catalase (60×10^3^), actin (43×10^3^), carbonic anhydrase (29×10^3^) and lysozyme (14×10^3^).

### Immunoblotting

1-D SDS-PAGE separated samples were placed in transfer buffer (12.5 mM Tris pH 8.8, 96 mM glycine, 20% methanol) and electrotransferred to PVDF membranes overnight using 100 mA/gel. Non-specific sites were blocked using 5% nonfat milk in Tween-20 TBS (TTBS) for 2 h, and then blots were washed in TTBS. Each blot was incubated with the primary antibody diluted in 2% nonfat milk in TTBS overnight, followed by the secondary antibody diluted in TTBS for 2 h. Blots were washed three times for 10 min in TTBS following each incubation. Following the final wash, blots were treated with ECL and exposed to x-ray film.

The primary antibodies used for immunoblotting were polyclonal rabbit anti- fibronectin (AB1954, 1∶4000; Millipore, Billerica, MA) and polyclonal rabbit anti-fibromodulin (sc-33772, 1∶200; Santa Cruz Biotech, Santa Cruz, CA). The secondary antibody used was HRP-conjugated anti-rabbit IgG (NA934, 1∶2000; GE Healthcare, Piscataway, NJ).

### LC-MS/MS

1-D gel lanes, representing 10-250×10^3^ *M*_r_, were cut into 12 equally sized 1 cm bands. Bands were destained twice using 200 µL 100 mM NH_4_HCO_3_/50% methanol for 5 min and then dehydrated using 200 µL 25 mM NH_4_HCO_3_/30% acetonitrile for 20 min followed by 100% acetonitrile for 1–2 min. Next, samples were dried for 3 min in a speed-vac concentrator. Reduction was performed using 50 µL 25 mM NH_4_HCO_3_/25 mM dithiothreitol at 56°C for 20 min. Alkylation was performed using 50 µL 25 mM NH_4_HCO_3_/55 mM iodoacetamide for 20 min in the dark. Samples were washed, dehydrated and dried as described above and then digested using 60 ng modified trypsin (Roche, Indianapolis, IN) in 15 µL 25 mM NH_4_HCO_3_ at 32°C overnight. Peptide extracts were reduced in volume to ∼10 µL in a speed-vac concentrator.

LC-MS/MS analysis was performed on a Micromass hybrid Q-TOF mass spectrometer with a nanoelectrospray source (Waters Corp, Milford, MA). Capillary voltage was set at 1.8 kV and cone voltage 32 V; collision energy was set according to mass and charge of the ion, from 14 eV to 50 eV. Chromatography was performed on a LC Packings HPLC with a C18 PepMap column (Dionex, Sunnyvale, CA) using a linear acetonitrile gradient and 200 nL/min flow rate.

Spectral peaks were extracted from raw data files using ProteinLynx 4.0 (Waters Corp) and default parameters. Peak lists (in PKL format) from the analysis of all 12 digested gel bands representing a single sample were concatenated using the Perl script merge.pl (http://www.matrixscience.com) and exported in MGF format. Peptide searches were performed using Mascot 2.0 (Matrix Science, London, UK) [49] running on a local server, with the following search parameters: tryptic digestion; one allowable missed cleavage; 0.2 Da tolerance for both precursor and fragment ions; 2+ and 3+ ions; fixed cysteine carbamidomethylation; variable methionine oxidation and NQ deamidation. Concatenated forward and reverse sequences from the NCBI Refseq rat protein database (updated 10.18.2006; 36,496 forward sequences) [50] were used for searching. This database was selected as it was rat specific, non-redundant and allowed decoy searching for the calculation of estimated false-positive rates. The threshold for positive protein identification was set using a 1% estimated false-positive rate, which corresponded to a probability based Mowse score of 32. Estimated false-positive rates and cut-off thresholds were calculated using previously reported algorithms [51] and scripts written in Mathematica 5.2 (Wolfram Research, Champaign, IL).

MS/MS spectra associated with local single peptide hits were subjected to additional validation using *de novo* peptide sequencing and MS-based BLAST searching, as follows. Relevant spectra were parsed from the concatenated MGF format data file and subjected to *de novo* analysis using PepNovo+3.1 beta, a previously reported probabilistic network-based sequencing algorithm [30]. Input parameters were identical to those used for Mascot database searching. Resulting candidate peptide sequences were submitted to a publically available MS-BLAST server (http://genetics.bwh.harvard.edu/msblast/) [52] using the nr95_clean database and default search parameters.

### Immunohistochemistry

Three additional age- and sex-matched Sprague Dawley rats were reserved for immunohistochemical validation of select proteins identified using LC-MS/MS. Laryngeal specimens were harvested and immediately embedded in optimum cutting temperature compound (Tissue-Tek; Sakura, Tokyo, Japan), frozen with acetone and dry ice, and stored at −80°C. The larynges were sectioned at an interval of 8 µm in the coronal plane using a cryostat (CM-3050 S; Leica, Wetzlar, Germany). Two adjacent coronal sections, containing the midmembranous vocal fold mucosa immediately anterior to the laryngeal alar cartilage, were selected from each animal for each marker of interest. The midmembranous mucosa was selected as it is an important tissue region for vocal fold oscillation; the laryngeal alar cartilage was selected as an anatomical landmark to ensure that all immunostained sections reflected a consistent anterior-posterior level in the coronal plane.

Frozen sections were fixed in 4% paraformaldehyde, washed with phosphate-buffered saline, and incubated with Block-Ace (AbD Serotech, Raleigh, NC) and 5% goat serum (Sigma) for 30 min to block nonspecific binding. Next, sections were incubated with primary antibody rabbit anti-collagen type I (ab34710, 1∶100; Abcam, Cambridge, MA), rabbit anti-fibronectin (LSL-LB-1027, 1∶300; Cosmo Bio, Tokyo, Japan), mouse anti-vimentin (M7020, 1∶200; Dako, Carpinteria, CA) or mouse anti-keratin Ka10 (MAB1605, 1∶200; Millipore) for 90 min, followed by secondary antibody rhodamine red conjugated goat anti-mouse or anti-rabbit IgG (1∶200; Jackson ImmunoResearch, West Grove, PA) for 60 min, with thorough wash steps between each incubation. Finally, slides were covered with antifade mounting medium with DAPI (Vectashield; Vector Labs, Burlingame, CA) and cover-slipped. Control sections stained with an isotype control or without the primary or secondary antibody showed no immunoreactivity (data not shown).

Immunostained images were captured using a fluorescent microscope (E-600; Nikon, Melville, NY) equipped with a digital microscopy camera (DP-71; Olympus, Center Valley, PA) at 100X magnification. Consistent exposure parameters were used for each marker to allow the direct comparison of fluorescent intensity across experimental conditions. Representative images were selected for presentation.
