# Correction: Cross-Sample Validation Provides Enhanced Proteome Coverage in Rat Vocal Fold Mucosa

**DOI:** 10.1371/annotation/369b65bc-2f17-461b-ae3f-90eb0778440d

**Published:** 2011-03-24

**Authors:** Nathan V. Welham, Masaru Yamashita, Seong Hee Choi, Changying Ling

Table S1 was erroneously omitted from the Supporting Information section. Please view Table S1 here: 

[^] 

