# Supplementary material for: Correction: Cross-Sample Validation Provides Enhanced Proteome Coverage in Rat Vocal Fold Mucosa
Source: PLoS One. 2011 Mar 24;6(3):10.1371/annotation/369b65bc-2f17-461b-ae3f-90eb0778440d. doi: 10.1371/annotation/369b65bc-2f17-461b-ae3f-90eb0778440d (PMC3068067; doi:10.1371/annotation/369b65bc-2f17-461b-ae3f-90eb0778440d)
Supplement: Supplementary file 1 [file pone.369b65bc-2f17-461b-ae3f-90eb0778440d.s001.pdf]

Table S1. Functional classification of proteins identified by LC-MS/MS. Data are drawn from three independent vocal fold mucosa samples using an identification threshold based on a 1% estimated false positive rate, removal of global single peptide hits, and removal of local single peptide hits derived from spectra that failed *de novo* peptide sequencing-based validation.

NCBI GI[a]	Protein name	Number of unique peptides[b]	Sequence Coverage (%)[b]	Theoretical M_r (x 10^3)	Theoretical p*I*
Circulatory system, blood proteins					
158138568	albumin	62	46	68.76	6.09
40445397	beta-glo	4	29	16.04	6.74
164448687	beta-globin	6	44	15.98	8.91
6978695	ceruloplasmin	3	3	120.84	5.34
6981010	hemoglobin alpha 1 chain	16	44	15.33	7.81
60678292	hemoglobin alpha 2 chain	16	44	15.28	8.45
17985949	hemoglobin beta chain complex	15	61	15.98	7.87
16758014	hemopexin	2	4	51.29	7.58
12831225	murinoglobulin 1	3	3	165.33	5.68
50657404	murinoglobulin 2	3	3	161.59	6.15
61556986	transferrin	21	26	76.40	7.14
6981684	transthyretin	2	17	15.75	5.77
Cytoskeletal proteins (microfilament, intermediate filament, microtubules) including nuclear envelope and epithelial keratins					
77993370	actin, alpha cardiac muscle 1	34	47	42.02	5.23
9506371	actin, alpha skeletal muscle	39	36	42.05	5.23
13592133	actin, beta, cytoplasmic	23	23	41.74	5.29
157823033	actin, beta-like 2	3	4	41.96	5.30
188536082	actin, gamma, cytoplasmic	29	30	41.80	5.31
109505063	actinin, alpha 2	7	4	106.74	5.34
57012436	keratin Ka10	16	9	56.51	5.10
57012432	keratin Ka11	9	7	52.60	5.01
51591909	keratin Ka13	6	9	47.73	4.85
56912233	keratin Ka14	24	15	52.68	5.08
51591903	keratin Ka15	29	19	48.87	4.80
56847618	keratin Ka16	18	11	50.78	5.05
47087085	keratin Ka17	26	14	48.12	4.97
68534953	keratin Ka18	6	13	47.76	5.17
42409519	keratin Ka19	23	27	44.64	5.21
57012446	keratin Ka22	7	11	50.21	5.09
57012440	keratin Ka28	3	5	45.84	4.79
57012370	keratin Ka38	10	2	48.94	4.99

120474989	keratin Kb1	23	7	64.76	8.04
114145409	keratin Kb15	6	4	57.61	8.04
57012352	keratin Kb18	8	5	62.17	7.59
57114290	keratin Kb2	7	5	69.13	7.58
57012362	keratin Kb35	5	2	60.44	7.53
57012358	keratin Kb36	14	5	60.39	8.17
57012368	keratin Kb39	14	9	57.25	5.48
57012360	keratin Kb4	27	21	57.67	7.52
50233797	keratin Kb5	11	8	61.93	6.90
40786432	keratin Kb8	14	11	54.02	5.83
57012372	keratin Kb9	6	5	61.76	8.65
160333156	LIM domain binding protein 3	5	11	30.99	9.17
6981262	nestin	3	1	198.7	4.31
13928744	transgelin	2	5	22.60	8.87
61557028	transgelin 2	2	10	22.39	8.41
78000188	tropomyosin 1, alpha	7	24	32.86	4.77
66730475	tropomyosin 2	7	23	32.96	4.63
8394466	troponin 1, type 2	2	4	21.33	8.86
58865337	troponin T3, skeletal, fast	3	6	30.36	6.47
11560133	tubulin, alpha 1	7	13	50.14	4.04
40018568	tubulin, beta 2	9	14	49.80	4.79
27465535	tubulin, beta 5	4	11	49.67	4.78
14389299	vimentin	15	32	53.73	5.06
DNA binding proteins					
109505801	histone H1c	10	19	33.16	10.76
18959218	histone H1d	9	22	21.99	11.10
6981004	histone H1f0	2	7	20.88	10.90
6981006	histone H1t	4	7	21.73	11.78
109505979	histone H2a	5	31	14.15	10.89
12025520	histone H2aa	5	32	14.28	11.02
109465265	histone H2ao	6	53	14.10	10.90
8393519	histone H2ay	3	6	39.50	9.79
12025526	histone H2bl	10	29	13.99	10.37
157818935	histone H3c2	3	13	15.39	11.27
12083635	histone H4b	3	19	11.37	11.36

Defense, stress, immune response proteins					
158138561	complement component 3	3	3	186.32	6.06
16924002	DJ-1 protein	3	7	19.97	6.32
11560024	heat shock 60 kDa protein 1	2	6	60.97	5.91
47059179	heat shock 70 kDa protein 1b	9	6	70.19	5.60
11177910	heat shock 70 kDa protein 2	6	6	69.53	5.44
25742763	heat shock 70 kDa protein 5	11	11	72.35	5.07
13242237	heat shock 70 kDa protein 8	12	12	70.87	5.37
148747365	heat shock 90 kDa protein 1, beta	2	3	83.28	4.97
62078619	immunoglobulin heavy chain, gamma	2	4	51.70	7.12
210032365	tumor rejection antigen gp96	3	6	92.77	4.72
Extracellular matrix proteins					
158711704	collagen, type 1 alpha 1	4	3	137.95	5.71
109509326	collagen, type VI alpha 1 chain precursor	7	8	108.81	5.21
54020664	decorin precursor	4	9	39.81	8.96
13929178	fibrillin 1	2	1	312.07	4.81
186972114	fibronectin 1	3	1	272.45	5.44
62659497	laminin gamma 1 chain precursor	2	1	177.39	5.09
6981204	matrix Gla protein precursor	2	23	12.04	9.27
16758080	procollagen, type I alpha 2	8	4	129.56	9.39
157817857	procollagen, type VI alpha 3	7	4	240.45	5.26
Membrane (cell, nuclear, mitochondrial) proteins					
6978501	annexin 1	4	16	38.83	6.97
9845234	annexin A2	9	23	38.68	7.55
55742832	annexin A4	2	5	35.89	5.43
9506497	clathrin heavy chain	2	1	191.60	5.50
20806141	solute carrier family 25, member 3	2	3	39.45	9.41
Metabolism and energy proteins					
40538860	aconitase 2, mitochondrial	2	4	85.43	7.87
14192933	aldehyde dehydrogenase 2	3	5	56.49	6.63
14192935	aldehyde dehydrogenase family 1, member A1	6	8	54.46	7.94
14010869	aldehyde dehydrogenase family 3, member A1	2	2	50.34	6.33
6978487	aldolase A	18	40	39.35	8.30
40538742	ATP synthase, H+ transporting, mitochondrial F1 complex, alpha subunit, isoform 1	10	19	59.75	9.22
54792127	ATP synthase, H+ transporting, mitochondrial F1 complex, beta subunit	12	15	56.35	5.18

20806153	ATP synthase, H+ transporting, mitochondrial F1 complex, delta subunit precursor	2	14	17.60	5.16
17157987	ATPase, Ca++ transporting, cardiac muscle, fast twitch 1	4	6	109.41	5.13
8392935	ATPase, Ca++ transporting, cardiac muscle, slow twitch 2	2	3	114.77	5.23
6978543	ATPase, Na+/K+ transporting, alpha 1	3	3	113.05	5.30
6978545	ATPase, Na+/K+ transporting, alpha 2	2	2	112.22	5.39
157817869	carbonic anhydrase 1	2	6	28.30	6.86
6978661	creatine kinase, muscle	15	27	43.02	6.58
6978725	cytochrome c, somatic	3	45	11.61	9.61
158186649	enolase 1, alpha	5	12	47.13	6.16
126723393	enolase 3, beta	5	11	47.01	7.08
158186722	fumarate hydratase 1	2	6	54.46	8.88
6980970	glutamate oxaloacetate transaminase 1	2	6	46.33	6.28
6980972	glutamate oxaloacetate transaminase 2	3	6	47.31	9.13
145275165	glutathione peroxidase 1	2	9	22.16	7.70
28933457	glutathione S-transferase, mu 2	5	17	25.70	6.91
8393418	glyceraldehyde-3-phosphate dehydrogenase	31	37	35.83	8.14
148747414	guanine deaminase	3	6	50.90	5.48
62079055	isocitrate dehydrogenase 2 (NADP+), mitochondrial	3	5	50.97	8.88
8393706	lactate dehydrogenase A	2	7	36.45	8.45
6981146	lactate dehydrogenase B	3	11	36.61	5.70
8394193	lipase, gastric	2	5	44.59	6.12
15100179	malate dehydrogenase 1, NAD	4	10	36.48	6.16
42476181	malate dehydrogenase, mitochondrial	3	11	35.68	8.93
20302061	mitochondrial ATP synthase, O subunit	2	10	23.40	10.03
19424338	mitochondrial trifunctional protein, beta subunit	2	7	51.41	9.50
11024650	myoglobin	6	38	17.16	7.83
16758348	peroxiredoxin 6	2	15	24.82	5.64
40254752	phosphoglycerate kinase 1	4	11	44.54	8.02
16757994	pyruvate kinase, muscle	6	10	57.82	6.63
18426858	succinate dehydrogenase complex, subunit A	2	4	71.62	6.75
12018252	transketolase	4	6	71.19	7.54
12621074	triosephosphate isomerase 1	4	20	26.92	6.45
55741544	ubiquinol-cytochrome c reductase core protein II	2	4	48.40	9.16

Cell motility, contractile/thick filament proteins					
109481744	myosin binding protein C, slow type	3	2	132.04	5.51
205830436	myosin heavy chain 2	61	22	223.32	5.61
6981234	myosin heavy chain 3	12	4	223.86	5.64
106879208	myosin heavy chain 4	33	14	222.88	5.58
186659510	myosin heavy chain 6	8	4	223.51	5.58
8393807	myosin heavy chain 7	8	4	223.08	5.64
109491001	myosin heavy chain 8	39	12	222.81	5.63
157823377	myosin light chain 6, alkali	3	19	16.96	4.46
13487933	myosin light chain fast, alkali	7	26	16.61	4.63
109470142	titin isoform N2-B	5	1	3703.80	6.10
83816939	alpha-1-inhibitor III	5	4	163.77	5.70
Protein fate (maturation, modification, trafficking, degradation)					
157822977	inter-alpha trypsin inhibitor, heavy chain 1	6	42	13.57	9.80
8393899	inter-alpha trypsin inhibitor, heavy chain 3	5	8	99.10	5.85
25282393	mast cell protease 2	4	17	27.10	8.18
201860276	mast cell protease 8	2	7	27.54	9.14
8394009	peptidylprolyl isomerase A	4	45	17.87	8.34
109497721	polyubiquitin	6	57	8.83	9.35
8393322	protein disulfide-isomerase A3	6	11	56.59	5.88
32563565	serine protease inhibitor 2a	2	5	68.22	5.31
13928716	serine protease inhibitor 2c	2	7	45.55	5.39
51036655	serine protease inhibitor alpha 1	8	11	46.12	5.70
42476287	transglutaminase 2, C polypeptide	6	11	76.94	4.95
6981710	tyrosine 3-monooxygenase/tryptophan 5-monooxygenase activation protein, eta	2	10	28.21	4.81
9507245	tyrosine 3-monooxygenase/tryptophan 5-monooxygenase activation protein, gamma	3	9	28.30	4.80
62990183	tyrosine 3-monooxygenase/tryptophan 5-monooxygenase activation protein, zeta	3	14	27.77	4.73
155369303	vacuolar protein sorting 13 homolog A	3	1	166.62	8.56
Signaling proteins					
6978477	alpha-2-HS-glycoprotein	3	9	38.00	6.30
157822131	anterior gradient homolog 2	2	4	19.87	9.04
8393206	cysteine and glycine-rich protein 1	2	8	20.61	8.90
157786744	dihydropyrimidinase-related protein 2	2	5	62.28	5.95
21728390	four and a half LIM domains protein 1, isoform 2	2	6	23.70	9.00

157819527	histidine triad nucleotide binding protein 1	2	17	13.78	6.36
8393693	laminin receptor 1	2	10	32.82	4.80
55742827	rho GDP dissociation inhibitor, alpha	3	15	23.41	5.12
84781723	tumor necrosis factor type 1 receptor associated protein	3	2	80.46	6.56
Protein translation/synthesis					
28460696	eukaryotic translation elongation factor 1, alpha 2	3	10	50.11	9.10
148747541	heterogeneous nuclear ribonucleoprotein U	3	3	87.73	5.92
62653926	ribosomal protein L12	2	15	17.85	9.35
14389297	ribosomal protein L19	2	13	23.47	11.48
157818939	ribosomal protein L23a	2	8	17.70	10.44
11968078	ribosomal protein L31	2	15	14.46	10.54
62642484	ribosomal protein L8	4	11	28.16	10.79
47087103	ribosomal protein S18	3	15	17.72	10.99
109483232	ribosomal protein S19	2	12	14.50	10.89
78126159	ribosomal protein S2	2	4	31.23	10.25
53850582	ribosomal protein S25	2	8	13.74	10.12
13592077	ribosomal protein S27a	8	36	17.95	9.68
57164151	ribosomal protein S3	2	7	26.67	9.68
13928986	ribosomal protein S8	2	12	24.21	10.32
Miscellaneous proteins					
19424346	common salivary protein 1	6	26	17.64	7.74
25282405	palate, lung and nasal epithelium carcinoma associated protein precursor	3	5	27.72	6.17

[a] National Center for Biotechnology Information General Identifier. Assignment is based on the closest isoform of the identified protein.
[b] Column entry represents the highest value across three independent samples.
